# Supplementary material for: SSR-Based Molecular Identification and Population Structure Analysis for Forage Pea (Pisum sativum var. arvense L.) Landraces
Source: Genes (Basel). 2022 Jun 18;13(6):1086. doi: 10.3390/genes13061086 (PMC9222440; doi:10.3390/genes13061086)
Supplement: Supplementary file 1 [file genes-13-01086-s001.zip › genes-1755733-supplementary.pdf]

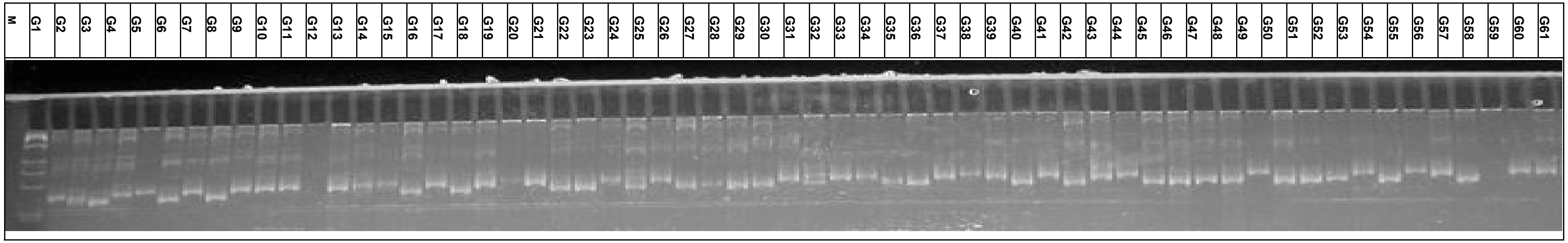

Figure S1. Gel images of SSR Marker within genotypes, band order from right to left: M (DNA Ladder), G1...and G61.
